# Kisspeptin as a marker for male infertility: a comparative study of serum and seminal plasma kisspeptin between fertile and infertile men

**DOI:** 10.1007/s10815-025-03644-w

**Published:** 2025-09-11

**Authors:** Nichamon Parkpinyo, Sirichet Anekpornwattana, Chantacha Sitticharoon, Somsin Petyim

**Affiliations:** 1 Reproductive and Biology Unit (RBU), Department of Obstetrics and Gynecology, Faculty of Medicine Siriraj Hospital, Mahidol University, Bangkok, Thailand; 2 Department of Physiology, Faculty of Medicine Siriraj Hospital, Mahidol, University, Bangkok, Thailand; 3 Center of Reproductive and Stem Cell Biology (CRSCB), Department of Obstetrics and Gynecology, Faculty of Medicine Siriraj Hospital, Mahidol University, 2 Prannok Road, Siriraj, Bangkoknoi, Bangkok, Thailand

**Keywords:** Kisspeptin, Male infertility, Semen parameters, Semen analysis

## Abstract

**Purpose:**

This study aimed to identify kisspeptin as a new marker for infertility in men with abnormal semen parameters by comparing serum and seminal plasma kisspeptin levels between fertile men and infertile men with normal and abnormal semen parameters.

**Methods:**

Fertile men (group A), infertile men with normal semen parameters (group B), and infertile men with abnormal semen parameters (group C) were recruited. Fasting venous blood was tested for kisspeptin, follicle-stimulating hormone (FSH), luteinizing hormone (LH), testosterone, insulin-like growth factor 1 (IGF-1), insulin, and glucose. Semen was collected by self-masturbation, and semen analysis was performed, then was tested for kisspeptin and testosterone.

**Results:**

Fifty-two men were included in the study (17 fertile men in group A, 18 infertile men in group B, and 17 infertile men in group C). Serum kisspeptin levels were significantly lower in fertile men (group A) as compared to infertile men (groups B and C) regardless to semen parameters (85.18 ± 20.47 ng/dL, 109.37 ± 28.64 ng/dL, and 108.70 ± 32.30 ng/dL respectively; *p* = 0.019). While seminal plasma kisspeptin levels were not significantly different (245.95 ± 67.12 ng/dL, 283.73 ± 119.82 ng/dL, and 312.99 ± 245.17 ng/dL, respectively; *p* = 0.48). There was no significant difference among groups for serum FSH, LH, testosterone, IGF-1, fasting insulin, fasting glucose, homeostasis model assessment of insulin resistance (HOMA-IR), and seminal plasma testosterone.

**Conclusion:**

Serum kisspeptin might be used as a more sensitive marker for male infertility rather than FSH and LH. However, the clinical application of kisspeptin in the treatment of male infertility requires further study.

## Introduction

Spermatogenesis is a complex process that occurs in seminiferous epithelium and epididymis. It involves the transformation of spermatogonial stem cells (SSCs) into immature sperm cells in testis, and into mature spermatozoa (sperm cells) throughout the epididymal duct. This process is tightly regulated and relies on the cooperation of Sertoli cells, Leydig cells, and the spermatogonia themselves. Cell communication, through endocrine signals, plays an important role in this process, for example, testosterone, gonadotropin-releasing hormone (GnRH), insulin-like growth factor 1 (IGF-1), and insulin-like peptide 3 (INSL3). Peptide hormones from the hypothalamus, pituitary gland, and other tissues, including follicle-stimulating hormone (FSH), luteinizing hormone (LH), insulin, inhibin B, and angiotensin II, play crucial regulatory roles in spermatogenesis by influencing testicular function and supporting the development and maturation of sperm cells [1]. It takes about 70 days to form the spermatocyte, and another 12–21 days are required for transportation [2]. Follicle stimulating hormone (FSH) and luteinizing hormone (LH) are pituitary hormones that are secreted from the anterior pituitary gland. FSH acts on the FSH receptor on Sertoli cells to support spermatogenesis, while LH acts on the LH receptor on Leydig cells to stimulate testosterone production. Testosterone plays a major role in several processes of normal spermatogenesis [3], for instance, spermatogonia mitosis, spermatocyte meiosis, and spermatocyte maturation [1]. Meanwhile, testosterone regulates gonadotropin secretion by negative feedback on GnRH secretion in the hypothalamus, which is an upstream control of gonadotropin secretion. However, it is not currently known whether the feedback is due to testosterone itself or its metabolites. Interestingly, recent evidence found estrogen and progesterone receptors in the arcuate nucleus of the hypothalamus [4–7]. Therefore, the physiological feedback of testosterone is probably mediated by aromatization to estradiol.

Kisspeptin, a 145 amino acid peptide, is encoded by the *KISS1* gene. After translation, kisspeptin is cleaved by proteolytic enzyme, resulting in a shorter peptide, the most common form is kisspeptin-54, followed by kisspeptin-14, kisspeptin-13, and kisspeptin-10, respectively [8]. Basically, kisspeptin acts on the kisspeptin receptor (KISS1R), the G protein-coupled receptor (Gpr54), which is found in several organs such as the gastrointestinal tract, liver, pancreas, pituitary glands, spinal cord, hypothalamus, and other regions of the brain [8]. Furthermore, kisspeptin receptors are found in sperm and reproductive organs, including the placenta, ovaries, and testes [8–15]. In sperm, kisspeptin receptors are mainly located in the head, around the neck, and the flagella, and their functions are still inconclusive [16–18].

Kisspeptin plays an essential role in regulation of the hypothalamic-pituitary–gonadal (HPG) axis. It works as an upstream control of gonadotropin-releasing hormone (GnRH) secretion [19–22], so it is essential for both pubertal development and fertility maintenance [8, 15]. In an animal study, Gpr54-knockout mice result in severe hypogonadism, infertility [23, 24]. Furthermore, many studies found that exogenous administration of kisspeptin-54 or kisspeptin-10 stimulates endogenous LH secretion in both men and women [25–30], while it has no further effect on growth hormone, prolactin, and TSH secretion [31]. Meanwhile, LH plays an indirect yet essential role in spermatogenesis by stimulating Leydig cells to produce testosterone, which is crucial for spermatogenesis. Moreover, gonadotropin-inhibitory hormone (GnIH) produced in the hypothalamus plays an important inhibitory role in the regulation of reproductive function by modulating the kisspeptin-GnRH-gonadotropin axis. GnIH can act directly on kisspeptin neurons, which suppress kisspeptin expression and release. This suppression results in reduced stimulation of GnRH neurons, which leads to decreased LH/FSH secretion. These events distort testicular histoarchitecture, impair testicular and adrenal steroidogenesis, lower spermatogenesis, and deteriorate sperm quality and function [13]. GnIH expression is upregulated by stress and energy deficiency.

Although the source of seminal kisspeptin is obscure whether from testes or accessory glands [32], the kisspeptin receptor has been found in spermatozoa. Study from Mei et al. found that both KISS1 and KISS1R mRNA were expressed by round spermatids beyond 1 month of the age of the mice. This finding may support that kisspeptin is important for sperm development [33]. Studies in mice have found that kisspeptin can induce spermatogonia proliferation and differentiation [34] and also has a role in sperm capacitation by participating in the calcium influx process [35]. However, there is little to know about the role of kisspeptin in human sperm function. An experimental study in human sperm found that the addition of kisspeptin in semen can induce transient sperm hyperactivation [16]. In healthy male volunteers, seminal plasma kisspeptin levels are positively correlated with sperm concentration, but there is no association with serum kisspeptin [36]. A previous study showed that serum kisspeptin levels in infertile men are significantly lower than those of fertile men [37], while another pilot study found that infertile men with abnormal semen parameters have lower kisspeptin levels than those with normal semen parameters [38]. However, there are some limitations to these studies. There is an uncertain definition of fertility, and there are some pitfalls in recruitment criteria. It also included men with hypogonadotropic hypogonadism that could affect serum kisspeptin levels and affect the results of the final study.

We hypothesized that serum and seminal plasma kisspeptin levels would be different between fertile and infertile men. For this reason, we conducted this study to compare both serum and seminal plasma kisspeptin levels among fertile men and infertile men with normal and abnormal semen parameters. In addition, our objective was to compare other serum profiles (FSH, LH, testosterone, IGF-I, FBS, insulin), and seminal plasma testosterone in fertile men and infertile men with normal and abnormal semen parameters. Moreover, this study was also to find the correlation between serum/seminal plasma kisspeptin levels and other parameters including serum profiles (FSH, LH, testosterone, IGF-I, FBS, insulin), seminal plasma testosterone. and different semen parameters.

## Materials and methods

### Study design and participants

The study protocol was approved by the Siriraj Institutional Review Board (Faculty of Medicine Siriraj Hospital, Mahidol University, Thailand) COA No. Si 075/2020.

This cross-sectional analytic study was conducted in the Department of Obstetrics and Gynecology, Siriraj Hospital, from January 24, 2020, to October 31, 2020. All methods were carried out following relevant guidelines and regulations. Informed consents were obtained from all subjects. The study intentionally recruited 18 fertile men (group A), who were proven fathers of children less than 1 year of age and after less than 1 year of regular sexual intercourse, and 38 infertile men, who did not produce a continuous pregnancy after regular sexual intercourse for at least 1 year. Subsequently, infertile men were classified according to results of semen analysis according to WHO 2010 criteria [39] into two groups: infertile men with normal semen parameters (group B) and infertile men with abnormal semen parameters (group C). All participants were informed to abstain from ejaculation for 2–7 days and fast for at least 8 h before an appointment day. A thorough history and physical examination were performed to exclude factors of female infertility (in infertile men), urogenital disease (such as cryptorchidism, varicocele, hydrocele, testicular mass, inguinal hernia), previous sexually transmitted infections, urogenital surgery, vasectomy, genital infections, current smoking > 15 cigarettes/day, and alcoholism. After recruitment, participants who were unable to ejaculate or had leukospermia (semen WBC > 1 million/mL) were later withdrawn from the study.

### Data collection

The baseline characteristics of the participants were collected, which included age, weight, height, previous illness, previous surgery, history of alcohol consumption, smoking, abstinence time, and number of previous children (in fertile group). The genital examination using orchidometer was performed by the same physician for all participants. The testicular volume was recorded in milliliters.

### Specimen collection

All participants were informed to abstain from ejaculation for 2–7 days and fast for at least 8 h before an appointment day. Venous blood was drawn on the morning of an appointment date and sent to the central laboratory for measurement of FSH, LH, testosterone, IGF-1, insulin, and glucose. A clotted blood tube was centrifuged at 1000 g for 10 min at room temperature, and an aliquot was collected, then preserved at − 80 °C before kisspeptin measurement.

Semen was collected by self-masturbation and sent to the infertility unit andrology laboratory for semen analysis. Sperm concentration and motility were assessed by computer-aided sperm analysis (CASA). Eosin-nigrosin and Diff Quik staining were performed to assess sperm viability and sperm morphology under microscopic visualization, respectively. Examination was performed at least 200 sperm cells per slide, and three replicas per sample were analyzed. All processes were performed by experienced technicians. The remaining semen was centrifuged at 300 g for 7 min at room temperature, and an aliquot was collected, then preserved at − 80 °C before measurement of testosterone and later measurement of kisspeptin.

### Homeostasis model assessment of insulin resistance (HOMA-IR) calculation

The assessment of insulin resistance by the homeostasis model was a product of fasting plasma glucose (mg/dL) and fasting insulin (uU/mL) divided by 405 [40].

### Kisspeptin measurement

Plasma and semen aliquots were diluted in a ratio of 1:4 and 1:8, respectively. Kisspeptin levels were measured using the KiSS-1 (112–121) amide/kisspeptin-10/metastin (45–54) amide (Human) EIA kit (Catalog number EK-048–56; Phoenix Pharmaceuticals, Inc., Burlingame, California, USA). The measurement protocol followed the manufacturer’s manual. EIA kits have 100% cross-reactivity to kisspeptin-54 and kisspeptin-10. The minimum detectable concentration is 0.06 ng/mL, and the linear range is 0.06 to 0.75 ng/mL. The intra- and inter-assay variations were < 10% and < 15%, respectively. The total seminal plasma kisspeptin was calculated by the production of semen volume and seminal plasma kisspeptin [36].

### Sample size calculation and statistical analyses

To detect a statistically significant difference in the mean of serum kisspeptin in fertile men in group A, infertile men with normozoospermia in group B, and infertile men with abnormal semen parameters in group C, sample size calculation, based on a previous study [38], required participants per 17 group to achieve 90% power with a significance level of 0.05. We include a sample size of at least 17 participants per group. We will recruit a total of 56 participants to account for potential dropouts, aiming for a lost follow-up rate of no more than 10%.

For demographic characteristics, continuous variables were analyzed and reported in mean ± S.D., and categorical variables were reported in percentage. The difference in means in each group was compared using a one-way ANOVA test, followed by the post hoc LSD test. Furthermore, the association between serum kisspeptin, seminal plasma kisspeptin, and other selected variables was explored using Pearson’s correlation. *P*-value < 0.05 was considered statistically significant. All statistical analyzes were performed with IBM SPSS statistics 26.0.

## Results

**Fig. 1:**
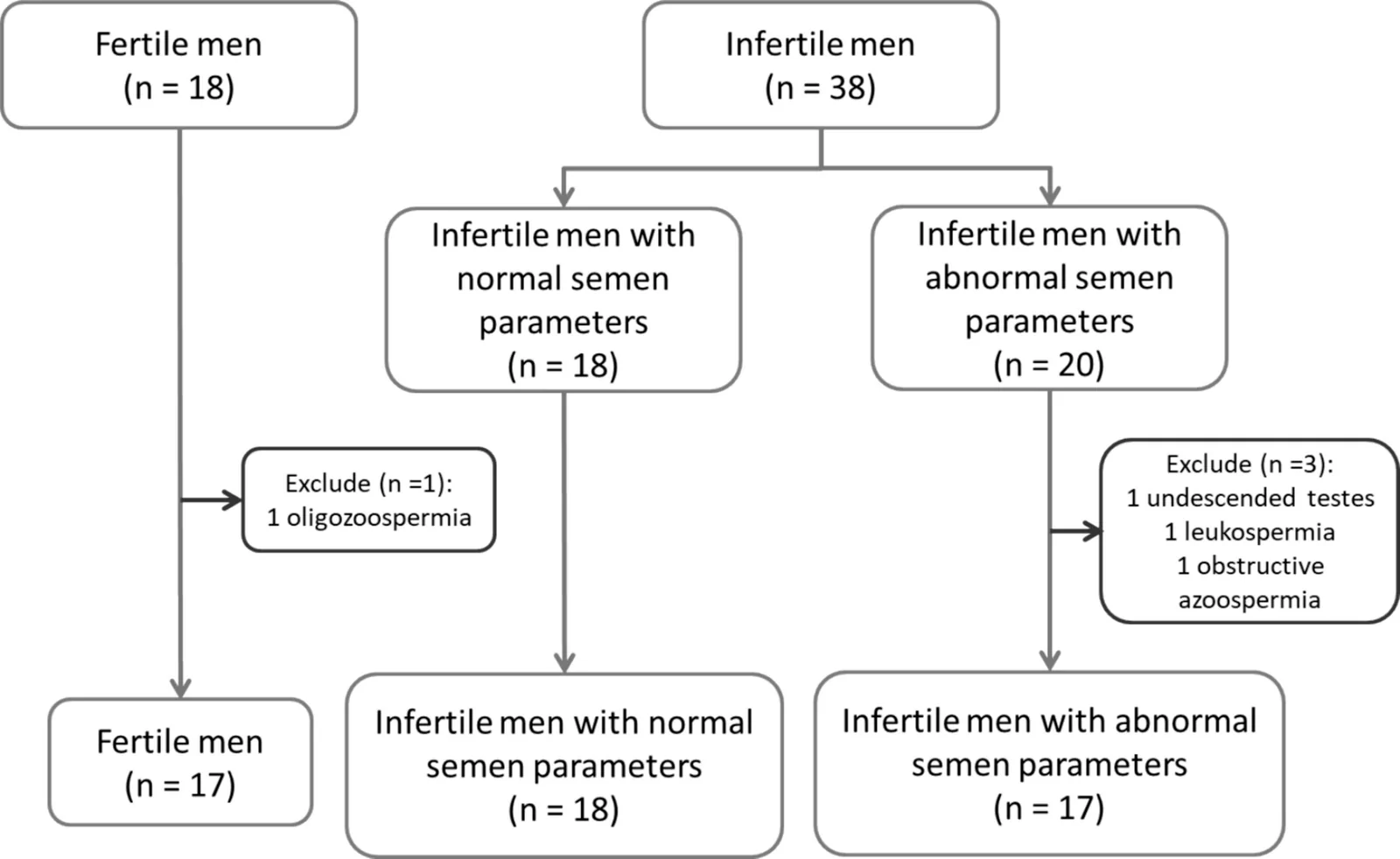
Recruitment and exclusion of participants in the study

In this study, 18 fertile men (group A) and 38 infertile men were initially recruited. A fertile man was excluded due to the presence of oligozoospermia, and after the results of the semen analysis were informed, he revealed that his wife achieved pregnancy after a more than 3-year attempt. Subsequently, infertile men were classified according to the results of the semen analysis according to the WHO 2010 criteria [39]. The infertile men were grouped as 18 infertile men with normal semen parameters (group B) and 20 infertile men with abnormal semen parameters (group C). Later, three men in group C were excluded from the study: one undescended testis, one leukospermia, and one obstructive azoospermia. Finally, there were 17, 18, and 17 men in groups A, B, and C, respectively (Fig. 1).

The mean age and BMI of all participants were 33.87 ± 3.91 years and 26.24 ± 3.91 kg/m^2^, respectively, which did not differ significantly between the groups. Most of them (57.7%) were overweight (BMI ≥ 25 kg/m^2^). The mean abstinence time was 4.02 ± 0.90 days. Among infertile men (groups B and C), there were no significant differences in duration of infertility; the mean was 4.20 ± 3.76 years (Table 1). In orchidometry examination, the mean testicular volume was significantly lower in group C compared to group A (13.62 ± 3.36 mL vs. 17.24 ± 3.67 mL, *p* = 0.009), while there was no significant difference from group B (16.06 ± 4.45 mL, *p* = 0.37) (Table 2).

**Table 1:** Demographic characteristics of the participants (mean ± S.D.)

	Group A Fertile men	Group B Infertile men with normal semen parameters	Group C Infertile men with abnormal semen parameters	*P* value
No. of patients	17	18	17	
Duration of infertility (years)		4.75 ± 4.19	3.62 ± 3.27	0.38
Age (years)	32.47 ± 3.52	35.17 ± 4.52	33.88 ± 3.28	0.13
Weight (kg)	76.22 ± 14.85	79.44 ± 15.34	80.41 ± 13.23	0.68
Height (cm)	172.60 ± 7.41	171.61 ± 6.41	174.41 ± 8.08	0.52
BMI (kg/m^2^) Underweight Normal Overweight Obesity	25.47 ± 4.10 0 (0%) 4 (23.5%) 5 (29.4%) 8 (47.1%)	26.84 ± 4.15 0 (0%) 2 (11.1%) 5 (27.8%) 11 (61.1%)	26.37 ± 3.53 0 (0%) 2 (11.8%) 4 (23.5%) 11 (64.7%)	0.58
Abstinence time (days)	3.94 ± 0.97	3.94 ± 0.94	4.18 ± 0.81	0.69

*P* values in the table are *P* value between three groups, which are the results of the one-way ANOVA test

**Table 2:** Clinical profiles of the participants, baseline hormones, and semen parameters (mean ± S.D.)

	Group A Fertile men	Group B Infertile men with normal semen parameters	Group C Infertile men with abnormal semen parameters	*P* value
No. of patients	17	18	17	
Average testicular volume (mL)	17.24 ± 3.67	16.06 ± 4.45	13.62 ± 3.36	0.027^+^
Serum FSH (IU/L)	4.18 ± 1.54	4.58 ± 1.82	4.90 ± 3.03	0.64
Serum LH (IU/L)	4.54 ± 1.16	4.68 ± 1.51	4.99 ± 2.09	0.72
Serum testosterone (ng/mL)	5.00 ± 1.59	4.22 ± 1.51	4.34 ± 1.40	0.27
Serum IGF-1 (ng/mL)	154.64 ± 48.91	149.02 ± 41.46	153.66 ± 44.10	0.92
Fasting serum insulin (uU/mL)	12.80 ± 9.23	13.92 ± 8.51	14.86 ± 13.67	0.85
Fasting blood glucose (mg/dL)	91.53 ± 9.74	91.56 ± 7.04	89.82 ± 8.84	0.80
HOMA-IR	2.98 ± 2.31	3.23 ± 2.12	3.29 ± 2.84	0.93
Serum kisspeptin (ng/dL)	85.18 ± 20.47	109.37 ± 28.64	108.70 ± 32.30	0.019*
Seminal plasma kisspeptin (ng/dL)	245.95 ± 67.12	283.73 ± 119.82	312.99 ± 245.17	0.48
Total seminal plasma kisspeptin (ng)	556.04 ± 289.18	739.55 ± 535.29	653.51 ± 329.03	0.91
Seminal plasma testosterone (ng/mL)	0.60 ± 0.30	0.72 ± 0.33	0.60 ± 0.37	0.51

*P* values in the table are *P* value between three groups, which are the results of the one-way ANOVA test

^+^*P*-value between group A, B = 0.37; A, C = 0.009; B, C = 0.068

^*^P-value between group A, B = 0.013; A, C = 0.016; B, C = 0.94

Serum kisspeptin levels were 85.18 ± 20.47 ng/dL in group A, 109.37 ± 28.64 ng/dL in group B, and 108.70 ± 32.30 ng/dL in group C. It represented significantly lower serum kisspeptin levels in fertile men (group A) as compared to infertile men (groups B and C) regardless of semen parameters. Although seminal plasma kisspeptin levels were 245.95 ± 67.12 ng/dL, 283.73 ± 119.82 ng/dL, and 312.99 ± 245.17 ng/dL in groups A, B, and C, respectively, which were not significantly different, there was no significant difference between the groups for serum FSH, LH, testosterone, IGF-1, fasting insulin, fasting glucose, HOMA-IR, and seminal plasma testosterone (Table 2).

**Fig. 2:**
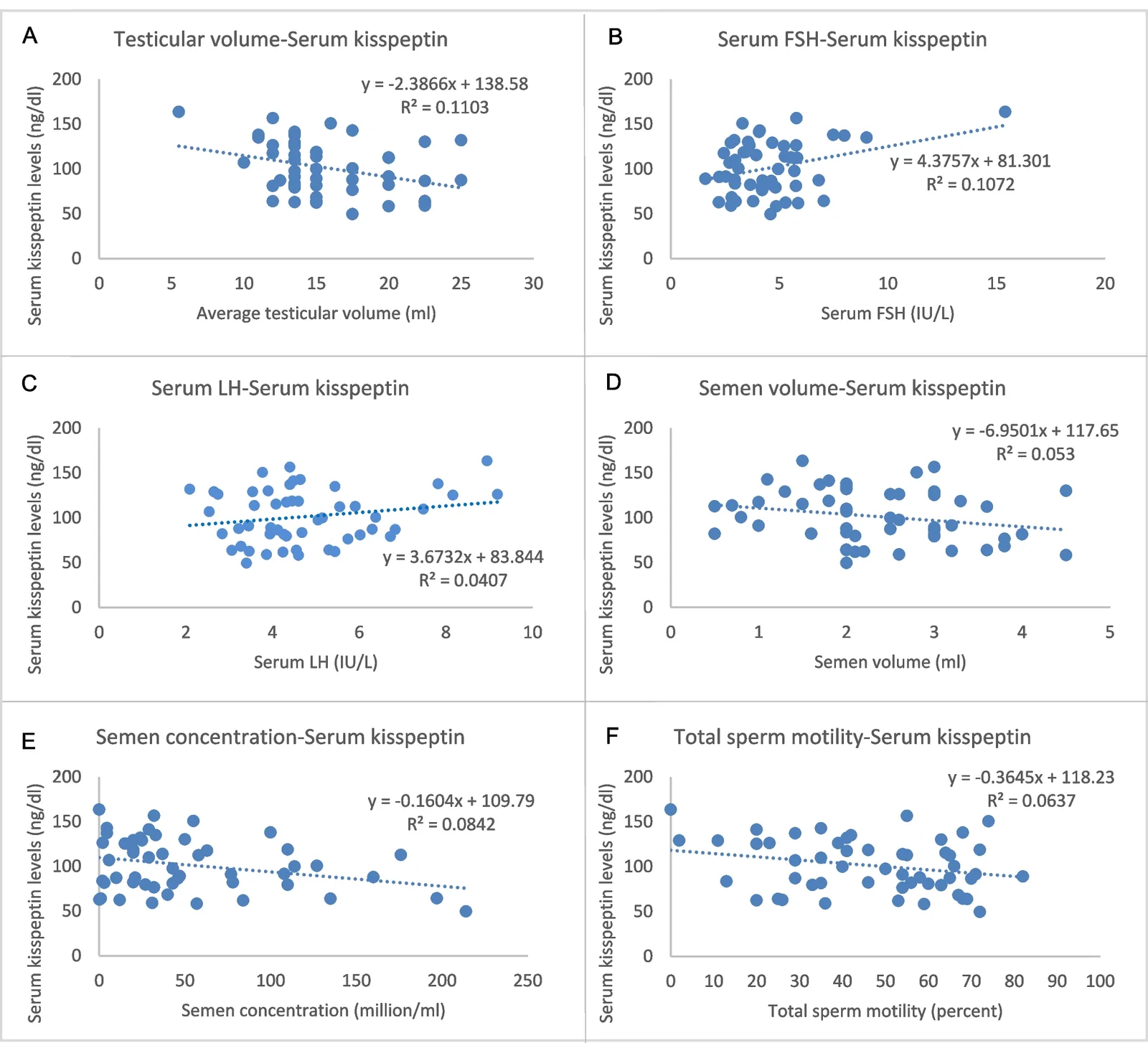
Correlations between serum kisspeptin levels and other variables; **A** mean testicular volume, **B** serum FSH, **C** LH, **D** semen volume, **E** sperm concentration, and **F** and total sperm motility

Among infertile men with abnormal semen parameters, which were subsequently classified according to WHO 2010 criteria [39], there are four oligoasthenozoospermic, six oligoasthenoteratozoospermic, three asthenozoospermic, three asthenoteratozoospermic, and one azoospermic men. In the association study, Pearson’s correlation showed that serum kisspeptin levels were negatively correlated with average testicular volume and sperm concentration (*r* − 0.332, − 0.290; *p* = 0.016, 0.037 respectively), while they were positively correlated with serum FSH (*r* 0.327, *p* 0.018), and there were no significant associations with other variables (Table 3; Fig. 2).

**Table 3:** Pearson correlation between serum/seminal plasma kisspeptin levels and other parameters

Variable	Serum kisspeptin level		Seminal plasma kisspeptin level	
	Correlation coefficient	*P* value	Correlation coefficient	*P* value
Age	0.130	0.36	− 0.171	0.23
Weight	0.134	0.34	− 0.118	0.40
BMI	0.231	0.099	− 0.058	0.68
Testicular volume	− 0.332*	0.016	− 0.032	0.82
Serum FSH	0.327*	0.018	0.060	0.68
Serum LH	0.202	0.15	− 0.033	0.82
Serum testosterone	− 0.145	0.31	− 0.131	0.35
IGF-1	0.096	0.50	0.211	0.13
FBG	0.061	0.67	− 0.282*	0.043
HOMA-IR	0.155	0.27	− 0.107	0.451
Serum kisspeptin	1	-	0.102	0.47
Seminal plasma kisspeptin	0.102	0.47	1	-
Semen testosterone	0.009	0.953	− 0.125	0.38
Semen parameters				
- volume	− 0.230	0.10	− 0.079	0.58
- concentration	− 0.290*	0.037	− 0.087	0.54
- total motility percentage	− 0.252	0.071	− 0.283*	0.042
- viability	− 0.145	0.30	− 0.481*	** < **0.01
- normal morphology percentage	− 0.018	0.90	− 0.024	0.87

**Fig. 3:**
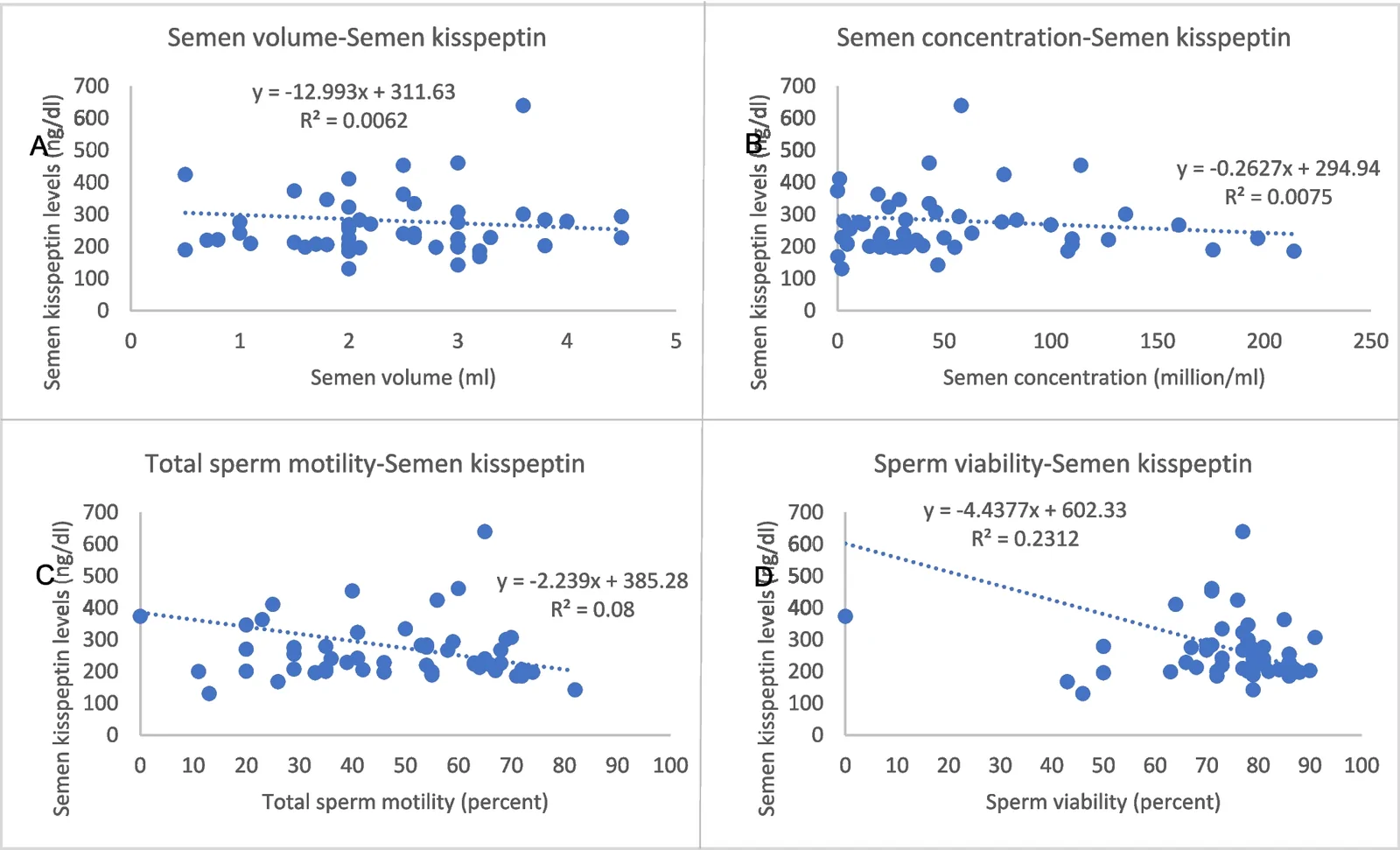
Correlations between seminal plasma kisspeptin levels and other variables; **A** semen volume, **B** sperm concentration, **C** total sperm motility, and **D** and sperm viability

For the seminal plasma kisspeptin level, it was negatively correlated with fasting blood glucose, percentage of total sperm motility, and sperm viability (*r* =  − 0.282, − 0.283, − 0.481; *p* 0.043, 0.042, < 0.01, respectively), while there were no significant associations with other variables (Table 3; Fig. 3).

## Discussion

This cross-sectional analytic study aimed to compare the serum and seminal plasma kisspeptin level among fertile men and infertile men with normal and abnormal semen parameters. Our hypothesis includes differences between groups, and kisspeptin might be correlated with male infertility. The study included 17 fertile men and 35 infertile men in the final analysis. Kisspeptins can be identified in both serum and semen in men of reproductive age. Interestingly, this is the first study to compare serum and seminal plasma kisspeptin levels between fertile and infertile men. This study also demonstrates the higher serum kisspeptin level in infertile men compared to fertile men.

In the study, serum kisspeptin levels were significantly lower in fertile men compared to infertile men regardless of semen parameters. To explain these results, kisspeptin plays a critical role in regulating the gonadotropin-releasing hormone (GnRH) pulse frequency and amplitude. This, in turn, controls the secretion of luteinizing hormone (LH) and follicle-stimulating hormone (FSH) from the anterior pituitary, which are essential for spermatogenesis and testosterone production. In infertile men, impaired testicular function may lead to low testosterone levels and disrupted spermatogenesis. The hypothalamus compensates by increasing kisspeptin secretion to enhance GnRH release in an attempt to stimulate LH and FSH production, thereby improving testicular function. In fertile men, the HPG axis is functioning optimally, with normal levels of GnRH, LH, FSH, testosterone, and spermatogenesis. Since the system is in balance, there is no need for excessive kisspeptin secretion to stimulate GnRH. This results in relatively lower circulating kisspeptin levels compared to infertile men. Recent evidence found that estrogen and progesterone receptors are present in KNDy neurons in the arcuate nucleus [4–7], but not in GnRH neurons. Despite scarce data, there is evidence that androgen receptors are also expressed in neurokinin B-containing neurons of the arcuate nucleus of the adult male rat [41]. Therefore, the physiological feedback of the sex steroids, including negative and positive feedback, is probably mediated by KNDy neurons [42]. However, some studies showed that KNDy neuronal fibers do not reach circulation. The retrograde tracing study with FluoroGold (FG) indicates that kisspeptin neurones are not in contact with fenestrated capillaries because peripheral administration of FluoroGold was found to label the majority of GnRH neurons but no kisspeptin neurons in female mice and rats [43, 44].

Although there is no evidence of the presence of an androgen receptor in the human hypothalamus, the physiological feedback loop of testosterone could be mediated by aromatization to estradiol and bind to estrogen receptors in the KNDy neuron [45]. Therefore, in the case of poor testicular function or male infertility, this feedback causes the increase of kisspeptin and FSH. For this reason, we hypothesized that kisspeptin is a more sensitive marker in poor testicular function than gonadotropins, as evidenced by higher levels of kisspeptin, but normal levels of FSH and LH in infertile men compared to fertile men. These seem to be supported by the negative correlation between serum kisspeptin levels with testicular volume and sperm concentration, and the positive correlation with serum FSH found in this study.

However, the results of this study are opposite to those of a previous study [37], which reported significantly lower serum kisspeptin levels in infertile men compared to fertile men. The difference in results may be explained by some possible reasons. First, there are differences in inclusion and exclusion criteria between studies. In previous studies, there are 53 men with hypogonadotropic hypogonadism in 150 infertile men (35.33%), which can lead to abnormal physiological feedback loops and low levels of levels of kisspeptin and gonadotropin in this group. Second, there is a more specific definition of fertility in our study, which narrows down the interval after his last child, confirming the curtained male fertility. Lastly, differences in kisspeptin measurement methods might affect the results, while there is currently no standard method for measurement.

As mentioned earlier, no previous studies have compared seminal plasma kisspeptin levels between fertile and infertile men. However, a study on healthy volunteers found a positive correlation between seminal plasma kisspeptin levels, sperm count, and total motile sperm. Notably, seminal plasma kisspeptin concentrations were approximately 60,000 times higher than serum levels [34]. Based on this finding, we initially diluted semen samples at ratios of 1:10^5^, 1:10^4^, 1:10^3^, and 1:10^2^ before measuring kisspeptin levels. However, the results were too low and fell outside the linear measurement range. Ultimately, we found that a 1:8 dilution ratio provided measurements within the linear range. The discrepancy in results may be attributed to differences in kisspeptin measurement methods and techniques. Despite these adjustments, our final analysis revealed no significant differences in seminal plasma kisspeptin levels between fertile and infertile men, regardless of semen analysis outcomes. Furthermore, no significant differences in other hormonal profiles were observed between the groups.

In the association study, we found that seminal plasma kisspeptin levels were negatively correlated with the percentage of total motile sperm and sperm viability, which could be explained by the probable compensatory response as in the association between serum kisspeptin and sperm concentrations. However, these results were in contrast to the previous study [36] and further association studies with a larger sample size might be required before reaching a conclusion.

This is a well-designed cross-sectional study and is the first study to show a significant increase in serum kisspeptin in infertile men, which is different from previous studies. Kisspeptin measurement in this study was performed using a reliable EIA kit by a single well-trained operator, and kisspeptin levels after dilution were all within the linear range of measurement as instructed by the manufacturer. So, the results were rather accurate. However, there are some limitations to this study. First, this is a single-center study in a small population, so generalization of the results could be limited. Second, the numbers of participants in this study are quite small to determine the difference in other serum and seminal plasma hormones. Additionally, subgroup analysis between each category of abnormal semen parameters could not be performed due to the limited number of participants. Further large studies might be needed to declare these questions, and GnIH measurement should be included in the further study.

## Conclusions

Our study found that serum kisspeptin levels were significantly higher in infertile men compared to fertile men, regardless of semen parameters. However, seminal plasma kisspeptin levels showed no significant differences between the groups. These findings suggest that kisspeptin may relate to male fertility and that serum kisspeptin might be used as one of the markers for male infertility. Nevertheless, the potential clinical applications of kisspeptin in the diagnosis and treatment of male infertility would require further investigation.

## Data Availability

All the data investigated and shown in this work is accessible from the authors upon an appropriate request.
